# Space Use by Crop‐Foraging Barbary Macaques When Crops Are Not Available

**DOI:** 10.1002/ece3.71916

**Published:** 2025-08-22

**Authors:** Elsa Minot, Alexandre Corbeau, Clara Tanvier, Juliette Menier, Latifa Silki, Zouhair Amhaouch, Pascaline Le Gouar, Nelly Ménard, Guillaume Péron

**Affiliations:** ^1^ CNRS, ECOBIO [(Ecosystèmes, biodiversité, évolution)] ‐ UMR 6553 University of Rennes Rennes France; ^2^ Département Des Parcs Nationaux et Des Aires Protégées Agence Nationale Des Eaux et Forêts Rabat Morocco; ^3^ CNRS LBBE UMR 5558, University of Lyon Villeurbanne France

## Abstract

We documented the influence of the section of the annual life cycle when crops were not available by tracking space use by two male Barbary macaques *Macaca sylvanus* that had access to the same cultivated area with cherry and walnut trees, adjacent to an oak forest near Ifrane NP, Morocco. Both individuals remained within a few kilometers of the orchards, even when there was no fruit available. They visited the orchards the most at the beginning of the study when walnuts were available and the herbaceous layer grew, leading to a peak in home range overlap, as predicted by the resource dispersion hypothesis. By contrast, they visited the orchards the least when cherries were available at the end of the study. These results indicated that the orchards and their vicinity hold resources that sustained the macaques outside of the cherry and walnut seasons, and confirmed that the wardens who were hired for this purpose efficiently protected the cherry crop.

## Graphical abstract

The spatial behavior of Barbary macaques around orchards during the winter. Using remote sensing data, we described the use of croplands by Barbary macaques ( *Macaca sylvanus* ) when fruit were no longer available in the orchards. Our study revealed that macaques remained in close proximity to the orchards throughout the winter but used them the least during the cherry season, when the orchards were protected by hired wardens.

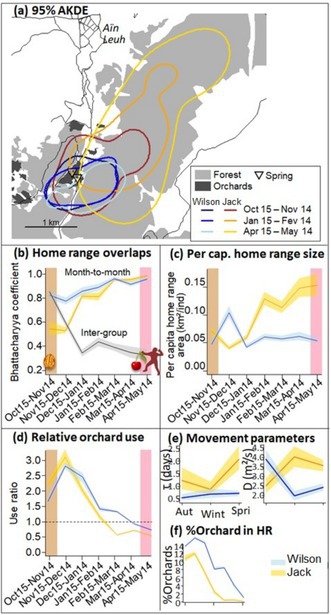

## 1 Introduction

The seasonality of crop availability could influence the balance between the costs and benefits of crop foraging for wildlife. If cultivated areas lacked food outside of the growing season, this constraint might be sufficient to prevent individuals from specializing on crops (Hill 2017). Alternatively, wildlife might perform seasonal movements to avoid the seasonal bottlenecks in food availability (Fox and Abraham 2017).

We described the movements of two male Barbary macaques *Macaca sylvanus* belonging to two different groups that we captured in the immediate vicinity of the same cultivated area near the town of Aïn Leuh, near Ifrane National Park in the Middle Atlas region of Morocco. This endangered primate is forest‐bound in our study region (Ménard, Rantier, et al. 2014). The forest is locally dominated by holm oaks *Quercus rotundifolia*. In this type of forest, the diet of the macaques is dominated by acorns and herbaceous plants, especially during the winter (Ménard and Vallet 1986; Ménard, Motsch, et al. 2014). However, orchards, and especially sweet cherry, have locally been planted right by the forest edge, in locations that used to be common land intended for livestock grazing. In the absence of physical barriers preventing the macaques from continuing to use the converted land, some macaque groups now forage on the fruit crop and on the herbaceous layer inside the orchards, leading to conflicts (Neves et al. 2023). Local stakeholders hire wardens who chase the macaques out of the orchards using loud noises, projectiles, and sometimes dogs (Neves et al. 2023). Only the cherry crop is protected that way. By contrast, the walnuts are not guarded by hired wardens (Neves et al. 2023) because landowners intend them mostly for private consumption. We used that as a quasi‐experiment regarding the effect of unguarded crops on macaques' space use.

This study lasted between October 2023 and May 2024, thus encompassing the mating period in autumn and the winter period of no crop availability, and allowing comparison with the early spring and late autumn. The cherry season started in April (flowers and green fruit). Walnuts were available from the start of the study to about mid‐November, at a time of the year when acorns represented a naturally‐occurring food resource for the macaques. For one of the two groups, the rest of the annual life cycle, from April to October, had already been documented (Neves et al. 2023; E. Minot, P. Le Gouar, Z. Amhaouch, S. Chollet, E. Demellier, A. Ernoult, N. Ménard, J. Menier, C. Tanvier, L. De Wever, G. Péron in prep.), and the winter season remained lesser studied.

The objective of the present Nature Note was to describe the whereabouts of the macaques when no fruit crop was available, to infer by comparison whether this period of the life cycle operated as a food availability bottleneck. Our elements of diagnosis were whether the home ranges became larger and involved more diffusive, less encamped movement when crops were not available, whether the individuals moved to a separate foraging ground in between periods of crop availability, and whether the home range overlaps decreased when crops were not available. Indeed, under the resource dispersion hypothesis (Macdonald 1983; Macdonald and Johnson 2015), we expected more home range overlap between our two study individuals when the crops were available because the crops represented a dense, localized, and sharable resource (Biswas et al. 2025).

## 2 Material and Method

We fit collar‐mounted GPS (Litetrack Iridium 150 Loteck with drop‐off system) to two adult males from two different groups, following the veterinary‐supervised capture protocol of which we elsewhere assessed the medium‐term impact on the captured individuals (Corbeau et al. 2024). The GPS were programmed to record one location every 30 min during the day.

Group “Depog” (called Group B in Corbeau et al. 2024) was made of 27 individuals (5 adult males, 5 adult females, 5 infants, and 12 immatures) and had already been monitored by Neves et al. (2023) and Minot et al. (in prep.). The equipped male from that group, named “Wilson”, yielded 6229 GPS fixes between October 15th 2023 and May 14th 2024. Group “Felix” was made of 46 individuals (12 males, 12 females, 5 infants, 17 immatures) and the equipped male, named “Jack”, yielded 5749 fixes over the same period.

For about 3 months (October, November, and May) we also tracked group Depog on foot, thereby making it possible to determine whether the movements of Wilson were representative of the movements of his group. After correcting the GPS error on Wilson's positions using a state‐space model (Péron et al. 2017), we estimated the distance between Wilson's position and the group centroid, recorded at the same time as the collar fix. We inferred that Wilson traveled with his group when that estimated distance was below 200 m, which corresponds to the spatial spread of a Barbary macaque group during diurnal activities. When the distance was > 200 m, we computed the duration of the episodes.

We also fit continuous‐time movement models and estimated the Ornstein–Uhlenbeck movement parameters (diffusion and home range crossing time), the weighted autocorrelated kernel density of use (AKDE), the home range area and core area (the 95% and 50% isopleths of the AKDE), and the home range overlap between the two individuals (Fleming and Calabrese 2017). We performed these analyses on a monthly basis by dividing the tracking data into month‐long segments, using the ctmm package for R with the recommended workflow (Calabrese et al. 2016).

We used the error option to inform the model‐fitting routine with data about the dilution of precision of each GPS fix, thereby making it possible to include all of our data with minimal filtering for outliers (Péron et al. 2017; Fleming et al. 2021). For the user equivalent range error, we provided a moderately informative prior with 95% of the weight between 1.5 and 15 m and a mean of 10 m (Fleming et al. 2021).

To perform statistical tests on the home range overlaps, we used the approximate confidence interval of the Bhattacharyya coefficient (Winner et al. 2018) using the overlap function in ctmm. The Bhattacharyya coefficient is weighted for the spatial variation in density of use, which means that the core home ranges (e.g., 50% isopleths) contributed more to the estimated overlaps than the peripheral regions (e.g., 95% isopleths).

**FIGURE 1:**
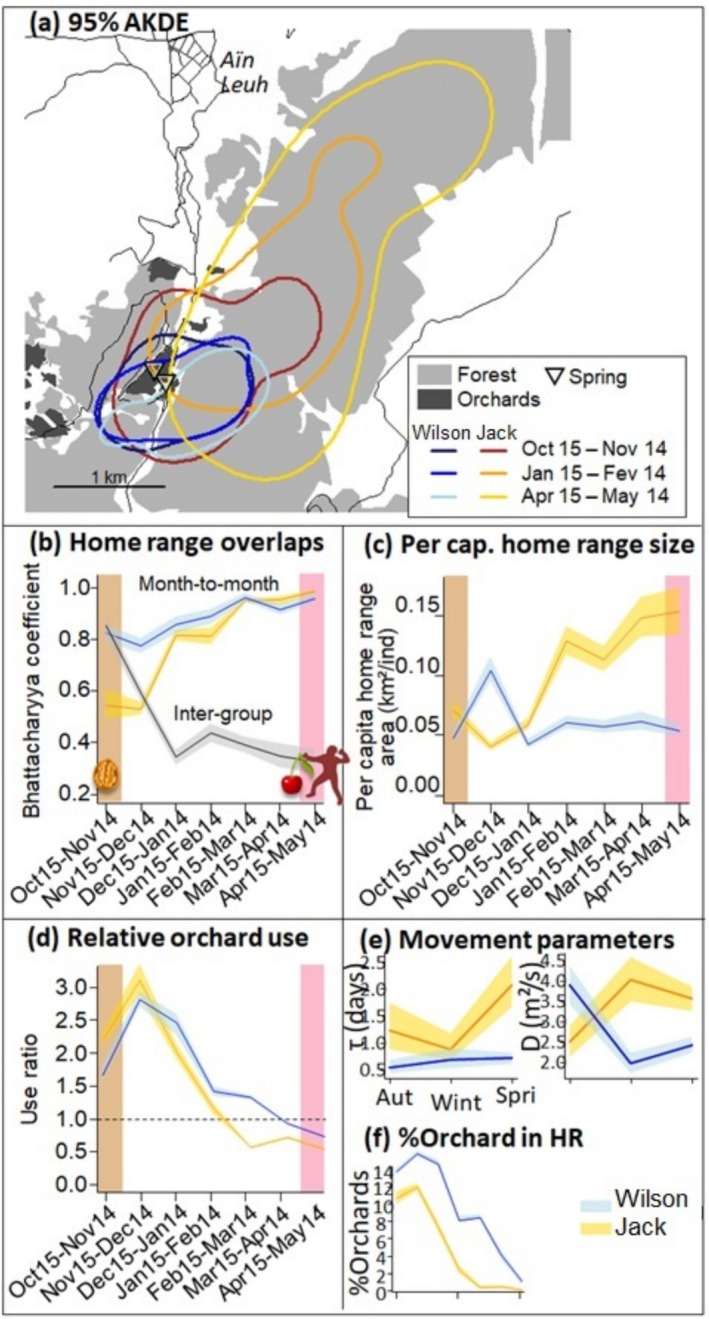
(a) 95% AKDE home ranges for three representative months and two groups of Barbary macaques that visited orchards near Aïn Leuh, Morocco, in 2023/2024. The thin black lines are tarred roads. Orchards further than 250 m from a forest edge are omitted. (b) Overlaps between successive monthly home ranges and monthly intergroup home range overlaps, weighted for the densities of use. Throughout, the shaded areas are 95% confidence intervals. (c) Monthly per capita home range sizes. (d) Monthly relative use of the orchards, which quantifies the perception bias for observers that would only record macaques encounters from the orchards. This ratio corresponds to how much more likely the orchards and a 200 m buffer around them were to be used by the macaques, compared with other landcovers in their home range, and after accounting for their relative frequency in the monthly 95% AKDE isopleth. (e) Estimated parameters from the continuous‐time Ornstein‐Uhlenbeck movement models fitted to the data. *τ* is the temporal autocorrelation time or home range crossing time. It is inversely proportional to the attraction for the home range centroid after an excursion. D is the diffusion parameter and represents the tendency to make excursions away from the home range centroid. The product D*τ is the movement variance which approximates the home range size. (f) Monthly proportion of orchards inside the 95% AKDE isopleth.

Lastly, we compared the AKDE probability of use in the orchards plus a 200 m buffer around the orchards, and the probability of use in the rest of the range, weighted by the areas of these two domains. If the ratio between the two was > 1, we concluded that the individual used the orchards more than expected from their availibility in the home range. Throughout, we did not report *p* values whenever the confidence intervals were far from overlapping in Figure 1. Otherwise, we performed Wald tests based on the standard errors and point values of the estimates.

Overall, these statistical methods are designed to accommodate differences in sampling schedules, missing data, GPS error rate, and movement rates, which otherwise could have biased the comparisons of space use between periods and groups (Fleming and Calabrese 2017). They are akin to an extrapolation of the long‐term space use around known used locations (Péron 2019).

## 3 Results and Discussion

For 75% of the time in October, 63% in November, and 94% in May, we estimated that Wilson travelled within 200 m of the group's centroid. The duration of the episodes during which Wilson's estimated position was > 200 m from the group centroid was 1 h 52 ± 29 min. Wilson always remained within the home range of group Depog during the study. Neither Wilson nor Jack dispersed from the groups in which we captured them.

Both individuals remained within 1–5 km of the orchards throughout the study, but exhibited different patterns of temporal variation in space use (Figure 1).

Wilson remained in the same small home range throughout the study; the overlap between successive monthly home ranges was 74% on average (Figure 1a–c: blue curves and confidence intervals). A possible explanation for the year‐round fidelity to the home range was that the orchards harbored an herbaceous layer, which was denser than inside the adjacent forest (E. Minot, P. Le Gouar, Z. Amhaouch, S. Chollet, E. Demellier, A. Ernoult, N. Ménard, J. Menier, C. Tanvier, L. De Wever, G. Péron in prep.). Mechanisms that favor the herbaceous layer inside the orchards even during the winter include irrigation and the reduced livestock grazing pressure. Herbaceous plants are a year‐round staple for Barbary macaques (Ménard and Vallet 1986; Ménard, Motsch, et al. 2014) that represent up to 50% of the diet even while foraging on crops (Neves et al. 2023). Herbaceous plants could therefore constitute an attractant for the macaques into the orchards. They could to some degree be considered as anthropogenic.

In other regions and in other species of macaques, the typical response to anthropogenic food availability was also a decrease in the per capita home ranges and daily path lengths (Izumiyama et al. 2003; Hansen et al. 2020; Jayapali et al. 2023; Xie et al. 2024). Overall, these elements suggested that, in macaques, a reduction in movement rates could be a general response to predictable, dense food availability.

However, Jack did not show the same pattern. Indeed, Jack extended its range into the forest to the north‐east, starting at a time that coincided with the end of the availability of walnuts in the orchards (circa Nov 15; Figure 1a). These patterns might correspond to circannual periodic movements, which our study period was too short to demonstrate, however.

Assuming that the individual home ranges represented the group home ranges, the per capita home range size was relatively constant for group Depog but increased for group Felix (Figure 1c). Overall, our data documented two different space use responses to the same anthropogenic resource. Elsewhere in the *Macaca* genus, there is at least one other instance in which the availability of anthropogenic food promoted more rather than fewer movements (Sha and Hanya 2013).

In the autumn and early winter, macaques used the orchards more than expected from their availability in the home ranges (Figure 1d). The macaques' bias for orchards, however, disappeared from February onwards (Figure 1d). During our study, the macaques visited the orchards the least when cherries started to be available (Figure 1f; Wald test Apr‐May vs. the rest: *p* < 0.001). Thus, our data confirmed the effectiveness of the wardens. The macaques visited the orchards the most when both walnuts and acorns were available and during one or 2 months after the walnuts had been depleted (Figure 1f). This meant that our study individuals preferred resources available in orchards over naturally occurring ones such as acorns. By contrast, in a different group of macaques that we monitored elsewhere and that did not have access to walnuts, acorns were an attractive resource (E. Minot, P. Le Gouar, Z. Amhaouch, S. Chollet, E. Demellier, A. Ernoult, N. Ménard, J. Menier, C. Tanvier, L. De Wever, G. Péron in prep.). Attracting the macaques back into the forest would thereby require a better attractant than acorns.

Regarding the role of surface water, in Jan‐Feb, the two permanent springs that the orchards harbored were barely included in Jack's 95% AKDE (Figure 1a: the two triangles vs. the golden shape). There was thus no evidence that the visits to the orchards by Jack during the winter were motivated by access to these two springs. At this time of year, the water content of the vegetation and maybe the snow appeared sufficient to meet the macaques' water requirements.

The Bhattacharyya coefficient of home range overlap between the two study individuals varied over time between 0.3 and 0.8 (confidence intervals in Figure 1b). The overlap was maximal in Oct‐Nov, that is, during walnut availability (Wald test Oct‐Nov vs. the rest: *p* < 0.001). In other words, the home range overlap was largest when a dense, localized, and sharable resource was available, which supported the resource distribution hypothesis. Similarly, in *Macaca mulatta* from Neilingding Island in Guangdong, China, the home range overlaps were the smallest in Jan‐Apr (Fan et al. 2024), which a priori was the time of year when the resource was the least abundant and most dispersed. Overall, the resource dispersion hypothesis appeared supported as a general framework to explain home range overlaps between macaque groups. The resource dispersion hypothesis also factors in the fact that group Felix had more individuals and thus required more space when it foraged for natural foods in winter and spring. Within the hypothesis, this mechanism helped explain the drop in overlap as a consequence of the extension of the home range of group Felix into the forest.

Lastly, the movement parameters that underlaid the temporal variation in home range size and home range overlap differed between the two individuals (Figure 1e). When one individual was encamped (small *D*, small *τ*), the other was diffusive (large *D*, large *τ*). In particular, Felix appeared encamped in the orchards in autumn when walnuts were available, whereas Wilson was the most diffusive at this time of year (Figure 1e). These movement parameter estimates suggested that when Felix was encamped in one place, Wilson was forced to move more, that is, displacement. A separate analysis of step selection corroborated the hypothesis of between‐group interference at medium distances and time scales (Péron et al. in prep.). However, the nature of the relationship between the two groups still remains to be specified through direct behavioral observations during intergroup encounters.

## 4 Conclusion

Using collar‐mounted GPS on Barbary macaques during a little‐studied period of their annual cycle, we cast a new light on the balance between the costs and benefits of crop foraging. Despite the lack of fruit crop for most of the study period, the orchards appeared to sustain Wilson (and most likely his group), throughout the study. Combined with data from Neves et al. (2023) and E. Minot, P. Le Gouar, Z. Amhaouch, S. Chollet, E. Demellier, A. Ernoult, N. Ménard, J. Menier, C. Tanvier, L. De Wever, G. Péron (in prep.) who documented the rest of the annual life cycle, we could conclude that for this group there was no seasonal bottleneck in food availability in the cultivated area. Thus, a potential action that stakeholders could undertake would be to remove the naturally occurring foods that sustain the macaques in and near the orchards when crops are not available: for example, mow the herbaceous vegetation.

However, Jack's data by constrast strongly suggested that removing the naturally occurring foods in and near the orchards was unlikely to be sufficient to fully prevent crop foraging. Indeed, Jack demonstrated a willingness to travel several kilometers between winter foraging grounds in the forest and autumn foraging grounds in the orchards. The increase in Jack's range after the walnut season, the decrease in home range overlap, and Jack's move toward the forest all indicated that Jack's group perceived and apparently successfully responded to a food availability bottleneck when crops were not available. The difference between Wilson and Jack might pertain to the larger size of Jack's group, meaning that this group required more food than the orchards could provide when crops were not available. Thus, instead of managing the benefits of crop foraging (Hill 2017), managing the perceived risks emerged as the better option (Calcagno et al. 2025), that is, continue and maybe extend the wardens' operations. Another risk‐based solution is the creation of bands of bare ground between the forest edge and the first trees of the orchards, that macaques would be reluctant to cross.

## Author Contributions

**Elsa Minot:** investigation (supporting), writing – original draft (supporting). **Alexandre Corbeau:** investigation (supporting), writing – review and editing (equal). **Clara Tanvier:** investigation (supporting). **Juliette Menier:** investigation (supporting). **Latifa Silki:** investigation (supporting), resources (supporting), writing – review and editing (supporting). **Zouhair Amhaouch:** resources (lead), writing – review and editing (supporting). **Pascaline Le Gouar:** supervision (lead), writing – review and editing (equal). **Nelly Ménard:** writing – review and editing (equal). **Guillaume Péron:** investigation (lead), methodology (lead), supervision (lead), writing – original draft (lead).

## Ethics Statement

This research was carried out in line with Moroccan laws. Our observations fulfilled the IPS Code of Best Practices for Field Primatology. Capture and data collection were approved by the Moroccan Forestry authorities and supervised by L.S.

## Conflicts of Interest

The authors declare no conflicts of interest.

## Data Availability

The datasets are stored at Movebank (www.movebank.org) under the ID 5910040565 (study name “ *Macaca sylvanus* —Péron—Morocco—GPS”).
